# CareConekta: study protocol for a randomized controlled trial of a mobile health intervention to improve engagement in postpartum HIV care in South Africa

**DOI:** 10.1186/s13063-020-4190-x

**Published:** 2020-03-12

**Authors:** Kate Clouse, Tamsin K. Phillips, Carol Camlin, Sandisiwe Noholoza, Phepo Mogoba, Julian Naidoo, Richard Langford, Martin Weiss, Christopher J. Seebregts, Landon Myer

**Affiliations:** 1 Vanderbilt University School of Nursing, Nashville, TN USA; 2 Vanderbilt Institute for Global Health, Vanderbilt University Medical Center, Nashville, TN USA; 3 Division of Epidemiology and Biostatistics, School of Public Health and Family Medicine, University of Cape Town, Cape Town, South Africa; 4 Center for Infectious Disease Epidemiology and Research, School of Public Health and Family Medicine, University of Cape Town, Cape Town, South Africa; 5 Bixby Center for Global Reproductive Health, Department of Obstetrics, Gynecology & Reproductive Sciences, University of California, San Francisco, CA USA; 6 Center for AIDS Prevention Studies, Division of Prevention Science, Department of Medicine, University of California, San Francisco, CA USA; 7 Jembi Health Systems, Cape Town, South Africa

**Keywords:** HIV/AIDS, Pregnancy, Postpartum, Women, Retention, Mobile health, mHealth, Smartphone, South Africa

## Abstract

**Background:**

South Africa is home to the world’s largest antiretroviral therapy program but sustaining engagement along the HIV care continuum has proven challenging in the country and throughout the wider region. Population mobility is common in South Africa, but there are important research gaps in describing this mobility and its impact on engagement in HIV care. Postpartum women and their infants in South Africa are known to be at high risk of dropping out of HIV care after delivery and are frequently mobile.

**Methods:**

In 2017, we developed a beta version of a smartphone application (app) - CareConekta - that detects a user’s smartphone location to allow for prospective characterization of mobility. Now we will adapt and test CareConekta to conduct essential formative work on mobility and evaluate an intervention - the CareConekta app plus text notifications and phone calls and/or WhatsApp messages - to facilitate engagement in HIV care during times of mobility. During the 3-year project period, our first objective is to evaluate the feasibility, acceptability, and initial efficacy of using CareConekta as an intervention to improve engagement in HIV care. Our second objective is to characterize mobility among South African women during the peripartum period and its impact on engagement in HIV care. We will enroll 200 eligible pregnant women living with HIV and receiving care at the Gugulethu Midwife Obstetric Unit in Cape Town, South Africa.

**Discussion:**

This work will provide critical information about mobility during the peripartum period and the impact on engagement in HIV care. Simultaneously, we will pilot test an intervention to improve engagement with rigorously assessed outcomes. If successful, CareConekta offers tremendous potential as a research and service tool that can be adapted and evaluated in multiple geographic regions, study contexts, and patient populations.

**Trial registration:**

ClinicalTrials.gov: NCT03836625. Registered on 8 February 2019.

## Administrative information

Note: the numbers in curly brackets in this protocol refer to SPIRIT checklist item numbers. The order of the items has been modified to group similar items (see http://www.equator-network.org/reporting-guidelines/spirit-2013-statement-defining-standard-protocol-items-for-clinical-trials/).

**Table:** 

Title {1}	CareConekta: study protocol for a randomized controlled trial of a mobile health intervention to improve engagement in postpartum HIV care in South Africa
Trial registration {2a and 2b}.	ClinicalTrials.gov NCT03836625; registered February 8, 2019
Protocol version {3}	Version 4.0; November 18, 2019
Funding {4}	National Institute of Mental Health and Office of Behavioral and Social Sciences Research (OBSSR), Office of the Director, National Institutes of Health (R34 MH118028)
Author details {5a}	Kate Clouse^1,2^* Tamsin Phillips^3,4^ Carol Camlin^5,6^ Sandisiwe Noholoza^4^ Phepo Mogoba^4^ Julian Naidoo^7^ Richard Langford^7^ Martin Weiss^7^ Christopher J. Seebregts^7^ Landon Myer^3,4^ * Corresponding author 1. Vanderbilt University School of Nursing, Nashville, TN, USA 2. Vanderbilt Institute for Global Health, Vanderbilt University Medical Center, Nashville, TN, USA 3. Division of Epidemiology and Biostatistics, School of Public Health and Family Medicine, University of Cape Town, Cape Town, South Africa 4. Center for Infectious Disease Epidemiology and Research, School of Public Health and Family Medicine, University of Cape Town, Cape Town, South Africa 5. Bixby Center for Global Reproductive Health, Department of Obstetrics, Gynecology & Reproductive Sciences, University of California, San Francisco, San Francisco, CA, USA 6. Center for AIDS Prevention Studies, Division of Prevention Science, Department of Medicine, University of California, San Francisco, San Francisco, CA, USA 7. Jembi Health Systems, Cape Town, South Africa
Name and contact information for the trial sponsor {5b}	National Institute of Mental Health 6001 Executive Boulevard Bethesda, MD 20892-9663
Role of sponsor {5c}	The funding sources had no role in the design of this study and will not have any role during its execution, analyses, interpretation of the data, or decision to submit results.

## Introduction

### Background and rationale {6a}

With more than 7 million HIV-infected people [1], South Africa is home to more people living with HIV than any other country in the world [2], and their national antiretroviral therapy (ART) program is the world’s largest [2]. South Africa adopted a “treat all” policy to provide ART to all HIV-positive people, regardless of CD4+ cell count, in 2016 [3]. Expanded ART availability has dramatically altered the health and quality of life of people living with HIV, with an estimated life expectancy gain of 11.3 years between 2003 and 2011 due to ART [4] and a 77% decrease in HIV transmission in stable serodiscordant couples [5]. However, the rapid scale up of the national ART program has put tremendous pressure on the limited resources of a public health sector that is hurrying to meet this immense need, and despite the widespread availability of ART, over 135,000 people died of AIDS-related causes in South Africa, according to data published in 2018 [1]. HIV and tuberculosis (usually exacerbated by HIV) are the top two causes of death among women ages 15–44 years in South Africa [6]. The potential for improved health through expanded ART availability will only be realized if individuals sustain engagement in HIV care.

Black African women of reproductive age (ages 20–34 years) are the population most heavily affected by the South African HIV epidemic, with 31.6% prevalence [7]. Expanded access to ART for all HIV-positive pregnant women has helped to reduce mother-to-child transmission of HIV to the point where near-elimination is now thought possible [8]. Despite this astounding achievement, there is mounting evidence that women who initiate ART during pregnancy may be at very high risk of loss to follow up (LTFU) [9–12], with re-engagement in routine HIV care after delivery a particular concern [13, 14]. The problem of patient LTFU within the public sector in South Africa has been well-documented [15, 16]. Our earlier research conducted in South Africa found that 25.2% of women pregnant at ART initiation were LTFU after one year, compared to 15.8% of men and 11.1% of non-pregnant women [10]. LTFU is higher after delivery than before: among women diagnosed with HIV during antenatal care in Johannesburg, 47.9% were lost within 6 months of delivery [13]; in Cape Town, disengagement was over twice as frequent in the postpartum period as during antenatal care [17].

A challenge to ensuring continuity of HIV care is that the fragmented healthcare system is not adapted for a mobile population. Patients considered LTFU actually may have switched facilities on their own as “silent transfers,” which typically are not recognized due to unlinked data across facilities [18–20]. This misclassification makes it very difficult to accurately assess the effectiveness of national ART programs. Mobility as a potential barrier to engagement in HIV care has been noted in Kenya [21, 22], Lesotho [23], Zambia [24], and the USA [25]. South Africa’s population is highly mobile; the most typical pattern is of circular, within-border migration between rural and urban areas, with the mobile individual keeping strong ties to the rural areas by sending remittances to their family and returning to the rural area for holidays and family events, and upon retirement, illness, or during lapses in employment [26]. Postpartum women in South Africa are likely to be a particularly mobile group, due to a tradition of returning to one’s rural home after delivery to receive care from family members [12, 27–29]. Our recent formative work at three sites in Johannesburg found that among 150 peripartum, HIV-positive women interviewed, 44% planned to travel around delivery - typically after delivery - to stay with family, sometimes leaving the infant in the care of family while the mother returned to work in Johannesburg [29]. With unlinked medical records and no way of tracking mobility, it is difficult to intervene appropriately to ensure continuity of HIV care for mother and infant.

Cell phones are as common in South Africa as they are in the USA [30], according to a 2015 Pew Research study that found that 89% of adults in South Africa owned a cell phone [30]. Our own research among pregnant, HIV-positive women attending public antenatal care in Johannesburg found that phone ownership was ubiquitous, phone sharing was uncommon (94% did not share), and the median time with the current phone number was 3 years [31]. Additionally, smartphone use is increasing rapidly; a 2015 survey found that half of South African respondents owned a smartphone [32]. While this does not represent complete saturation, South Africa is rapidly adopting increasingly sophisticated mobile phones; thus, it is a very appropriate country in the African region to develop and test mobile health (mHealth) interventions, and lessons learned there will be relevant throughout the continent as other countries similarly adopt new technologies. mHealth interventions, particularly short message service (SMS) interventions, have been shown to improve adherence to ART [33, 34] and patient retention [35, 36]. Global positioning system (GPS) technology has been used successfully to track subject location in several studies, mostly in developed countries [37–39]. A few HIV-related studies that used mobility-tracking wearable devices to assess mobility have found acceptability and feasibility among young men who have sex with men [40] and transgender women [41] in the USA, demonstrating the relevance and utility of mobility-tracking studies in other settings and populations.

CareConekta is a novel smartphone app that uses GPS to identify the user’s location coordinates to meet two primary functions: (1) observe mobility prospectively, and (2) to allow the participant to locate nearby ART facilities using current location and a pre-loaded list of health facilities in South Africa, searchable through Google Maps. The app was devised by the study investigators (Drs Clouse, Phillips, and Myer) in parallel with a series of focus group discussions with potential users, developed as a beta version at Vanderbilt University Medical Center by Dr Martin Were and team, and then redeveloped and adapted by Jembi Health Systems in Cape Town. Regular “heartbeat” signals from the phone’s GPS system provides the participant’s current location geocoordinates, which are made “fuzzy” by transmitting a random location within one kilometer (km) of each exact location to protect privacy. Data charges related to the app are reverse-billed to the study, so that participants do not pay extra for cellular data. Knowledge of traveling participants allows the investigators to intervene in real time; thus, if the participant in the intervention arm travels more than 50 km outside the study area for more than 7 days, the app will automatically notify the participant of available care facilities near her new location. Additionally, study staff may contact the traveling participants through phone calls and/or WhatsApp to assist in linkage to care.

Through this 3-year study, we seek to assess the feasibility, acceptability and initial efficacy of using CareConekta to improve engagement in HIV care among postpartum women in South Africa.

## Objectives {7}

### Aim 1

Aim 1 is to characterize mobility among South African women during the peripartum period and its impact on engagement in HIV care.

#### Aim 1a

Aim 1a it to use GPS location data from CareConekta and spatial analysis to characterize peripartum mobility within the complete observational cohort (*n* = 200), including the frequency, distance, and timing.

#### Aim 1b

Aim 2b is to assess the association between mobility and engagement in HIV care for mother (retention in care and viral suppression 6 months after delivery) and infant (completion of routine early infant diagnosis).

### Aim 2

Aim 2 is to evaluate enhanced CareConekta as an intervention to improve engagement in HIV care.

#### Aim 2a

Aim 2a is to assess the acceptability and feasibility of the standard and enhanced app, with a focus on identifying user preference and implementation outcomes.

#### Aim 2b

Aim 2b is to evaluate the initial efficacy of the enhanced CareConekta intervention, using notifications and staff contact to improve engagement in HIV care, by assessing the association between study arm and engagement in HIV care at 6 months postpartum.

## Trial design {8}

This is a single-center, open-label, parallel-arm, randomized controlled trial to assess the feasibility, acceptability, and initial efficacy of using a novel smartphone application to improve engagement in postpartum HIV care in South Africa.

## Methods: Participants, interventions and outcomes

### Study setting {9}

The study site for participant enrollment is the Gugulethu Midwife Obstetric Unit (MOU), based at the Gugulethu Community Health Center near Cape Town, South Africa. The MOU serves Gugulethu as well as the surrounding informal settlements, and is often attended by women who come from the Eastern Cape to have their babies in Cape Town, resulting in travel to and from the Eastern Cape during pregnancy and postpartum [42]. The local antenatal HIV prevalence is high (~ 30%), and the mother-to-child HIV transmission rate is estimated at 2–4% [43]. The MOU team has helped to deliver HIV care and treatment services in this setting since 2003 and has a history of successful ART service delivery and research in partnership with the provincial government. Members of the study team have conducted studies at the MOU since 2006 [44].

### Eligibility criteria {10}

Eligible subjects are adult (≥ 18 years) women living with HIV who are in the third trimester of pregnancy (≥ 28 weeks gestation) and are willing to be randomized. Subjects must be able to speak and understand isiXhosa and/or English. Basic smartphone-level literacy must be demonstrated by asking the participant to read aloud the following sentence in either English or isiXhosa: “I can look up a new clinic on the phone.” Last, eligible participants must currently own a smartphone that meets the technical requirements of the app and be willing to opt in to installation of the app on her personal phone and to agree to mobility tracking. The specific technical requirements of the app include (1) a touchscreen, (2) capable of accessing the Internet, (3) operating system Android, version 5.0 or later, (4) cellular service is provided by one of the four major networks in South Africa (Cell-C, MTN, Telkom, or Vodacom), (5) capable of using GPS to show current location, and (6) requires charging the battery less than twice a day, on average. In our preliminary work, nearly 90% of smartphones of women approached for study participation used the Android system [45].

Late pregnancy was selected in order to allow for a 9-month total follow-up time within the time constraints of a 3-year project. In South Africa, most women present for their first antenatal visit around 20 weeks gestation or later [13, 46, 47], and attendance for antenatal visits is generally high [13, 17], so we do not believe there will be substantial bias introduced by enrolling women later in pregnancy. isiXhosa is the predominant language in the region of the study site, and English also is widely spoken, so we do not anticipate excluding potential participants due to language. Basic smartphone-level literacy will be required of participants in order to assess the impact of messages. However, we anticipate that most women who own a smartphone already have a basic level of smartphone literacy. We acknowledge that women who own a smartphone may be different to those who do not, in terms of employment, education, or other factors.

### Who will take informed consent? {26a}

Written informed consent is obtained by a study team member fluent in isiXhosa and English who has completed Human Subjects Protections and Good Clinical Practice certification. All women who meet the eligibility criteria and are interested in participating in the study will undergo written informed consent, which will be available in English and isiXhosa. The consent form will confirm the eligible woman’s understanding of the study procedures and her informed willingness to participate. The key messages of the informed consent process will include (1) a description of the study goals and procedures, (2) voluntary participation in the study, (3) confidentiality and privacy with use of the app and study data, (4) the right to withdraw at any time, and (5) the standard of care received at the MOU or any other healthcare facility will not differ according to participation in the study.

Once the consent form has been explained to the participant, any questions she may have will be answered. If the participant agrees to provide consent for participation, she will indicate this with her signature on the consent form. If she does not wish to participate or does not provide her consent for all the activities, she will not sign the consent form and will not be enrolled. A second copy of the form will be provided for her to sign and keep, if requested. The study staff member who administered the form will provide his or her name, signature, and date as a witness to the signing. Signed informed consent forms will be stored in binders within a locked office and only accessible by study staff.

### Additional consent provisions for collection and use of participant data and biological specimens {26b}

The informed consent form specifically requests permission for installation of the app, location tracking, data collection, medical record review (participant and infant), one blood draw, and re-contact by study staff.

## Interventions

### Explanation for the choice of comparators {6b}

In order to assess CareConekta’s ability to track participant location, the CareConekta app will be installed for all study participants - regardless of study arm. This also will allow all participants to passively search for new clinics using the facility finder. To assess the potential of using real-time location data in order to improve engagement in HIV care, participants in the intervention arm will receive enhanced follow up when they travel.

### Intervention description {11a}

Participants in the control arm (*n* = 100) will receive standard CareConekta, which will track their mobility and allow the participant to look up new ART facilities upon request. Participants in the intervention arm (*n* = 100) also will receive standard CareConekta with the following additions:

- Push notifications prompting the participant to open the app to view nearby ART facilities when they have traveled > 50 km from the study site for > 7 days.
- Phone call(s) and/or WhatsApp message(s) from study staff when they have traveled > 50 km from the study site for > 7 days. The study-staff calls and messages will ask about medication supply and will provide assistance with nearby facilities, if requested.

### Criteria for discontinuing or modifying allocated interventions {11b}

The additional communication included in the intervention arm will be discontinued at the participant’s request.

### Strategies to improve adherence to interventions {11c}

The intervention will be delivered only if the participant has met the travel threshold described above. No additional participant behavior is required in order to receive the intervention. To ensure staff fidelity of the intervention, push notifications are standardized, and a brief script has been developed (in English and isiXhosa) for items to be addressed in the phone calls and/or WhatsApp messages to participants. If app “heartbeats” are not received, participants will be contacted to troubleshoot technical problems, regardless of study arm.

### Relevant concomitant care permitted or prohibited during the trial {11d}

Subjects are ineligible for study participation if they are currently involved in another research study that may impact the study outcomes. Routine clinical care will continue as usual for all participants.

### Provisions for post-trial care {30}

During and after the trial, participants will continue to receive routine care at the clinic of their choice.

## Outcomes {12}

This study will collect data by the following three methods:

- GPS location (mobility) data collected through the CareConekta app
- Participant responses to questionnaires at the enrollment and follow-up visits, and during a brief postpartum interim phone call
- Post-study review of participant electronic and paper medical records

### Primary outcome: Mobility within the study period

We will use data from all 200 participants to describe mobility within a complete observational cohort. In order to assess mobility comprehensively, we will use multiple data sources: time and date-stamp data and geographic location data from CareConekta, participant self-report during questionnaires, and electronic medical records during the file review.

### Secondary outcome 1: Impact of mobility on engagement in HIV care in the absence of the intervention

Limiting the analysis to only the 100 participants in the control arm, we will explore the impact of mobility on engagement in HIV care in the absence of intervention by using GPS location data and engagement in HIV care data retrieved through medical record review. To assess engagement in HIV care, we will report retention in care, which will be assessed at the 6-month postpartum point and defined as no documented HIV-specific contact with any healthcare facility (including medication pick-ups) for > 3 months. We also will report viral suppression 6 months after delivery based on the viral load test closest to the 6-month period, using thresholds of ≤ 50 and ≤ 1000 copies/ml. Additionally, we will report vertical HIV transmission and completion of the routine 10-week infant polymerase chain reaction (PCR) test.

### Secondary outcome 2: Acceptability and feasibility of CareConekta

Acceptability and feasibility data will be collected among all 200 participants. Those in the intervention arm will receive additional questions related to the enhanced app features. The outcomes selected for analysis correspond with the Technology Acceptance Model for Resource-Limited Settings (TAM-RLS) framework, which was designed to draw attention to end-user acceptability as a predictor of mHealth technology adoption in low-resource settings [48].

### Secondary outcome 3: Initial efficacy of the intervention

The overall efficacy analysis will include participants in both arms (*n* = 200). Engagement in HIV care will be defined as for the measure in “Secondary outcome 1”. Study arm assignment is the primary exposure of interest.

### Participant timeline {13}

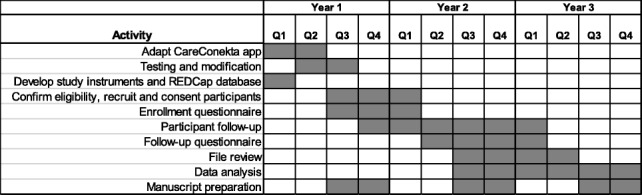



### Sample size {14}

We will enroll 200 participants in total. Sample size was not formally estimated for this pilot trial. The sample size was reached based on the feasible enrollment from the study site within the study timeline. Approximately 4000 women seek antenatal care at the MOU annually; ~ 30% of these are HIV-positive, about 1200 per year, or 100 per month. Our formative work at the study site showed that over half of these women own smartphones. If we conservatively estimate that 30% of all pregnant, HIV-positive women seeking antenatal care each month will be eligible and interested, this equals 30 participants enrolled per month. We anticipate that it will take approximately 7 months to reach our enrollment target of 200 participants.

### Recruitment {15}

Each week during the study recruitment period, study staff will review the list of upcoming appointments at the study site to assess who may be eligible for study participation. Pregnant women attending routine antenatal care services at the Gugulethu Community Health Center will be approached by trained study recruiters and given brief information about the pilot study. If they are interested, they will be screened to ensure they meet the eligibility criteria, using an eligibility checklist. If eligible, they also will be shown a demonstration of CareConekta and its functionality prior to the informed consent process.

## Assignment of interventions: allocation

### Sequence generation {16a}

The randomization sequence will be generated in R with a 1:1 allocation ratio within randomly permuted blocks to ensure balanced allocation to the intervention and control arms.

### Concealment mechanism {16b}

Randomization assignment will be concealed in sequentially numbered, opaque sealed envelopes until opened after enrollment with the participant.

### Implementation {16c}

The sequence will be generated by a statistician independent of the study team. The randomization assignment envelopes will be created by an individual at the University of Cape Town independent of the study team following the generated sequence. After enrolling a participant, the site staff will select the next consecutive sealed envelope to reveal study arm assignment.

## Assignment of interventions: Blinding

### Who will be blinded {17a}

Due to the open-label nature of this study, no blinding will be used.

### Procedure for unblinding if needed {17b}

Due to the open-label nature of this study, no blinding will be used.

## Data collection and management

### Plans for assessment and collection of outcomes {18a}

Mobility data will be collected throughout the study using the GPS tracking feature of the CareConekta app.

For recording participant characteristics, several study measures have been used previously by the study investigators in similar populations at the study site and in other areas of South Africa. In addition, we are using the following widely used, validated measurement scales:

- ART adherence: 3-item self-report measure (Wilson, et al.) [49]
- Alcohol use: Alcohol Use Disorders Identification Test-Concise (AUDIT-C) [50]
- Drugs: Question 1 from Drug Use Disorders Identification Test (DUDIT) [51]
- Depression: Patient Health Questionnaire-4 (PHQ-4) [52]
- Intimate partner violence: World Health Organization Violence Against Women (WHO VAW) measurement [53]
- Life events [54]
- Patient-healthcare provider relationship [55]
- Perceived availability of support [56]
- Social impact scale [57]

Study forms are available by request to the investigators.

### Plans to promote participant retention and complete follow up {18b}

In order to maximize the potential of re-contacting each participant and accessing medical records, we will collect identifying information on a separate paper-based linkage form, including contact information, name, study number, clinic record number, phone number, national identification number, address, and date of birth, and we will also update this form with infant information, when available. As is standard practice for research studies at the study site, the participant may also provide details for an alternative contact person in the event that we are unable to reach the participant after multiple attempts. No details about the participant will be shared with the alternative contact; study staff simply will ask that the participant return to the clinic when she can. For the interim phone call and follow-up visit, a minimum of three contact attempts by phone and/or home visit will be made for each participant. If participant mobility data cease to transmit at least once per day, the study team will follow up with the participant to find out if there are problems with her phone.

### Data management {19}

All hard copies of study documents, including signed consent forms, linkage forms, and logs, will be stored in locked cabinets in the study office with only the study team having access to the data. For increased protection, the linkage form and signed consent forms will be stored separately from other study documents.

Enrollment and follow-up questionnaires are designed in Research Electronic Data Capture (REDCap) and will be administered electronically using the REDCap mobile app. REDCap is a secure, web-based application designed to support data capture for research studies, providing (1) an intuitive interface for validated data entry; (2) audit trails for tracking data manipulation and export procedures; (3) automated export procedures for seamless data downloads to common statistical packages; and (4) procedures for importing data from external sources [58]. Range checks for numerical values and prompts about skipped questions are built into the REDCap database. The data will be collected with the study interviewer/fieldworker - who is fluent in isiXhosa and English - during a face-to-face interaction with the participant. The study coordinator will review all participant data to ensure completion. The Principal Investigator (PI) also will monitor the study safety data on an ongoing basis. The encrypted REDCap data servers will be housed at Vanderbilt University Medical Center. Participant questionnaire data and medical record data will be exported from REDCap, and mobility data from CareConekta will be imported from .csv files to SAS for data analysis.

The transmission and storage of sensitive participant information will follow best practices regarding smartphone app confidentiality. All information collected by CareConekta, including location, device ID, and time stamp, will be encrypted and transmitted to secure study servers using the secure https protocol. Time-stamped GPS coordinates will be stored in a longitudinal history. Electronic mobility data will be stored on a back-end storage system at the South African Medical Research Council (MRC) that meets Health Normative Standards Framework guidelines, which call for restricted access, and the separation of patient demographics and health data. The user reference (unique ID or demographic information of the patient) is separated from the user’s cell number and phone ID, as well as separated from the location information for security and purposes.

### Confidentiality {27}

Certainly, the concept of tracing an individual’s personal location is one that must address substantial protections of human subjects. First, our formative research with potential users in focus group discussions specifically addressed issues of privacy regarding a mobility-tracing app and notifications. For both issues, we found high acceptability [59].

While we feel that our formative research clearly indicates initial acceptability of mobility tracing and notifications, we have designed this work with a strong commitment to offering the highest level of protection of our participants’ privacy and confidentiality. First, the location data of interest are at the macro-level, showing movement between towns, cities, or regions, not at the micro-level, showing specific whereabouts. Thus, to protect participants’ privacy, the transmitted location information will be made “fuzzy” by transmitting a random location within 1 km of each participant’s exact location. Any participant’s exact location within that radius will be unknown. All information collected by the app, including location, device ID, and time stamp, will be transmitted to secure study servers using the secure https protocol. The participant’s numerical study ID number will be used during installation; no identifiable participant information will be recorded in the app.

There is always the possibility of a breach of confidentiality of study materials when conducting research. While this is acknowledged, there is very low likelihood because of the precautions that will be taken to protect confidentiality. Study staff will be trained on the expectations that they are not to disclose any information collected in the study to anyone outside the study team. All identifying information associated with the study participants will be maintained in locked storage cabinets and password-protected computers that themselves are kept in locked rooms or cabinets. All participants will be encouraged to contact the clinic staff, PIs, or other staff to report any undesirable conduct associated with the study. These reports will be brought to the attention of the PI.

Another risk to participants is that an HIV-positive status may be discerned through disclosure of participation in the study. However, we will do everything possible to minimize the risk of disclosure. No communication, whether on the phone or by text message, will identify the participant as HIV-positive. Importantly, among participants in the intervention arm who receive notifications of new facilities and phone calls and/or WhatsApp messages, the study staff will not use HIV-specific language. To avoid the possibility of inadvertent disclosure, ART facilities will only be referred to generically as “clinics” and any reference to ART will be to “tablets,” a common term for any medication that we demonstrated was acceptable in an earlier mHealth study in South Africa [46].

Risk to subjects will be minimized by (1) training research staff in the ethical conduct of research; (2) strict protection of confidentiality by detailed standard operating procedures and on-site monitoring; (3) careful handling of sensitive data, procedures, and processes in place to ensure data is securely stored and transferred; and (4) referral of participants to appropriate social and health services when necessary. The study site is well-connected with the social worker and psychology services at the site and with local non-governmental organizations providing counseling and support. While the potential risks to subjects are low, any social harm will be reported to the PI and a serious event will be reported to the Vanderbilt University and University of Cape Town Institutional Review Boards (IRBs) within 48 h of becoming aware of the event. Should any respondent experience discomfort or distress from participation in the study, we will provide the participant with the appropriate referral needed for follow up and care.

Risk to subjects from participation in the study is minimal. All possible precautions will be taken to ensure complete confidentiality and protection of study participants. Given the minimal risk, the overall benefit of the study outweighs the risks.

### Plans for collection, laboratory evaluation, and storage of biological specimens for genetic or molecular analysis in this trial/future use {33}

We will not collect blood or other biological specimens during this study. In routine care in this setting, viral load tests are to be repeated every 3 months in pregnancy and every 6 months during breastfeeding, so we expect that most women will have a routine viral load completed before 6 months postpartum. To avoid additional blood draws for the participant, at the follow-up visit, the study staff will check electronic health records to determine the most recent viral load test. If the participant has had no viral load test within 3 months prior to the 6-month study follow-up visit, she will be referred for a blood draw for routine viral load testing at the clinic. Study staff will not conduct the blood draw and blood will not be stored.

## Statistical methods

### Statistical methods for primary and secondary outcomes {20a}

#### Primary outcome: Mobility within the study period

To assess the primary outcome of interest, we will report the number of participants who travel during the study period (defined as > 50 km for > 7 days), over the denominator of all participants. Among those who travel, we will report the median and interquartile range number of trips during the study period and will determine timing and frequency of travel as it relates to delivery date, which will be obtained from the participant questionnaire and/or medical records. Median and interquartile range for duration of travel will be reported in days, and we will also assess *temporicity* (frequency of trips * duration of trips). Reasons for travel will be obtained from the enrollment and follow-up questionnaire and will be grouped by theme, such as family, work, housing, and education.

For a more detailed assessment of mobility, we will use ArcMap to geographically place all locations in which participants were recorded during the study period. We will plot origin and destination locations on a map of South Africa, and neighboring countries (if necessary). We will connect origin and destination locations using ArcMap’s XY-to-line tool, and will use a spherical calculator to estimate the distance between origin and destination. The geographic data will be used in conjunction with the novel metrics of mobility developed by Dr Camlin, which are reflective of the complex forms of mobility in sub-Saharan Africa. For example, we will apply geopolitical boundaries to location codes so that measures of internal migration and localized mobility within and across those boundaries can be estimated, such as inter-provincial and intra-provincial mobility. We also will map mobility flow according to origin and destination pairs, classified by geopolitical categories. We will thereby demonstrate how geographic data can be used to characterize the mobility of populations - providing a vital alternative to survey measures - in order to measure the impacts of that mobility on care cascade outcomes, with broad applicability to the measurement of its impact on other health outcomes. We also will conduct a secondary analysis to validate whether self-reported anticipated travel matches data collected from the CareConekta app.

#### Secondary outcome 1: Impact of mobility on engagement in HIV care in the absence of the intervention

We will limit the analysis to the control arm only, to explore the impact of mobility on engagement in HIV care in the absence of intervention. We will report baseline participant characteristics as collected on the enrollment questionnaire. We will report proportions and 95% confidence intervals for categorical variables and medians and interquartile ranges for continuous variables.

To assess engagement in HIV care, we will report retention in care, which will be assessed at the 6-month postpartum point and defined as no documented HIV-specific contact with any healthcare facility (including medication pick-ups) for > 3 months. We also will report viral suppression 6 months after delivery, using thresholds of ≤ 50 and ≤ 1000 copies/ml. Additionally, we will report vertical HIV transmission and completion of the routine 10-week infant PCR test.

We will fit a series of models of mobility metrics on outcomes. For one, we will use a dichotomous exposure of mobility (any travel > 50 km: yes/no), we will use Cox proportional hazard regression models to estimate the association of mobility on these outcomes, and will produce adjusted hazard ratios and 95% confidence intervals for retention in care and achieving viral suppression. We also will identify other predictors of engagement in HIV care. We will produce crude Kaplan-Meier curves for time to loss to follow up and time to viral suppression, by mobility group. Additionally, we will perform sensitivity analyses to explore how the estimated effect size changes when using varying cutoff points to define mobility.

#### Secondary outcome 2: Acceptability and feasibility of CareConekta

We will report proportions and 95% confidence intervals for categorical variables and medians and interquartile ranges for continuous variables. Open-ended questions will be reviewed for key themes. Themes will be abstracted and quantified.

#### Secondary outcome 3: Initial efficacy of the intervention

We will use Cox proportional hazard regression models to estimate the association of study arm assignment with these outcomes, and will produce adjusted hazard ratios and 95% confidence intervals for retention in care and achieving viral suppression, by study arm. We also will identify other predictors of engagement in HIV care. We will produce crude Kaplan-Meier curves for time to loss to follow up and time to viral suppression by study arm. In a secondary analysis, we will perform an as-treated analysis using self-reported data on actually reading the notifications and/or WhatsApp messages or staff phone calls, to analyze the effect of receiving the intervention.

### Interim analyses {21b}

No interim analyses are anticipated, and no stopping guidelines have been established, due to the low risk of participant harm during this study.

### Methods for additional analyses (e.g. subgroup analyses) {20b}

Anticipated analyses are as described above.

### Methods in analysis to handle protocol non-adherence and any statistical methods to handle missing data {20c}

The primary study analysis will be an intention-to-treat analysis based on actual random allocations. Secondary per-protocol analyses will be performed if there has been protocol non-adherence.

The nature of missing data will be assessed and, where appropriate, sensitivity analyses will be conducted using multiple imputation.

### Plans to give access to the full protocol, participant-level data and statistical code {31c}

This study is registered on ClinicalTrials.gov (NCT03836625), so the protocol is publicly available. De-identified participant-level data and statistical code will be available by request of the study investigators.

## Oversight and monitoring

### Composition of the coordinating center and trial steering committee {5d}

Due to the relatively small nature of the study team, additional coordinating centers and committees will not be convened.

### Composition of the data monitoring committee, its role and reporting structure {21a}

As this is a pilot study, a data safety and monitoring committee is not required and will not be convened.

### Adverse event reporting and harms {22}

We anticipate that the potential for adverse events (AEs) and serious adverse events (SAEs) related to study participation is low. However, we will document, investigate, and follow up all potentially study-related AEs, in accordance with IRB guidelines and sponsor policies. All reports will be made in writing to the study sponsor program officer.

Additionally, there is always the potential for social harms to participants of clinical trials, particularly when the clinical trial is limited to individuals with HIV. Any social harm will be reported to the PI and considered an AE and/or SAE and reported according to the aforementioned schedule. Should any respondent experience discomfort or distress from participation in the study, we will provide the participant with the appropriate referral needed for follow up and care.

### Frequency and plans for auditing trial conduct {23}

Study data and procedures will be regularly reviewed by the study team. No formal study audits are planned.

### Plans for communicating important protocol amendments to relevant parties (e.g. trial participants, ethical committees) {25}

In the unlikely event that we must revise our consent form to reflect changes to the study protocol, study staff will re-contact participants to bring them back to the clinic to be re-consented. Home visits will be made, if necessary. The public study record available on ClinicalTrials.gov also will be updated at the time of IRB approval of the new documents.

## Dissemination plans {31a}

We anticipate numerous manuscripts resulting from this study. Manuscripts will be written by members of the study team, and we will also welcome predoctoral and postdoctoral trainees affiliated with our institutions to lead secondary manuscripts to facilitate capacity building. Study results will also be shared at national and international HIV meetings and with the municipal, provincial, and national departments of health in South Africa.

## Discussion

Engagement in HIV care through the pregnancy and postpartum period is a substantial challenge to the success of the South African national ART program, as well as other countries in the region. This innovative study seeks to provide critical information to understand mobility during a specific, limited time period and to assess the potential impact of mobility on engagement in HIV care, while also providing a novel tool for improving engagement.

This study is among the first to track participant mobility in real time and use that information to intervene to improve engagement in HIV care. Collecting and using participant location information through smartphones is not without critics. Beginning with our formative research, we have closely considered user privacy. In focus group discussions, after first describing mobility tracking, we explicitly asked, “What do you think about a cell phone recording location so that researchers can use it? Would you want to do this?” and found high acceptability [59]. Most commercial smartphone apps collect a host of user data, often including GPS data; in our case, we want the potential user to make an informed decision to enroll, to safeguard the data as much as possible, and to use the data to address critical gaps in research about engagement in HIV care.

This study will provide considerable practical information about the feasibility of implementing smartphone-based applications in a resource-limited setting. We acknowledge that women who own a smartphone may be different to those who do not, in terms of employment, education, or other factors. Besides the additional financial resources needed to provide phones, we want to be able to assess CareConekta in existing phones for maximal impact. If women already have a smartphone (estimated at over half of the population), providing a second phone for study purposes is redundant and burdensome. For women who do not already own a smartphone, we are concerned with the ethical implications of providing such a highly desired item as a smartphone in terms of possible coercion for study participation, the impact it may have on relationships between participants and partners and/or friends, and the potential for disclosure for participating in an HIV-related study.

Our study represents a participant population accessing care at one research site only. The proposed site is long-established as a qualified research site under the direction of our co-investigators. Engagement in postpartum HIV care is a problem among the patient population, as similarly demonstrated in studies throughout South Africa, and women are known to travel during the peripartum period, most frequently to the Eastern Cape Province of South Africa. We believe that the study site and the population it serves is representative of other public antenatal clinics in peri-urban South Africa.

If we are successful in demonstrating the feasibility, acceptability, and initial efficacy of CareConekta, our goal is to assess it in a larger randomized, controlled trial in different regions. CareConekta offers tremendous potential as a research and service tool and can be adapted and evaluated in multiple geographic regions, study contexts, and patient populations.

## Trial status

This is protocol Version 4.0; dated 18 November 2019. Enrollment began on 9 December 2019 and will continue approximately through 30 June 2020.

## Data Availability

De-identified data will be made available to researchers outside of the study team, by request.

## Abbreviations

AE: Adverse event
ART: Antiretroviral therapy
GPS: Global positioning system
HIV: Human immunodeficiency virus
IRB: Institutional review board
LTFU: Loss to follow up
mHealth: Mobile health
MOU: Midwife obstetric unit
NIH: US National Institutes of Health
SAE: Serious adverse event
TN CFAR: Tennessee Center for AIDS Research
